# Effects of *Lactobacillus plantarum* TWK10-Fermented Soymilk on Deoxycorticosterone Acetate-Salt-Induced Hypertension and Associated Dementia in Rats

**DOI:** 10.3390/nu8050260

**Published:** 2016-05-02

**Authors:** Te-Hua Liu, Jiachi Chiou, Tsung-Yu Tsai

**Affiliations:** 1 Department of Food Science, Fu Jen Catholic University, New Taipei City 24205, Taiwan; r19901217@hotmail.com; 2 Shenzhen Key Lab for Food Biological Safety Control, Food Safety and Technology Research Center, Hong Kong Polytechnic University Shenzhen Research Institute, Shenzhen 518000, China; jiachi.amber.chiou@polyu.edu.hk

**Keywords:** hypertension, fermented soymilk, reactive oxygen species, neuronal cell degeneration, dementia

## Abstract

Oxidative stress resulting from excessive production of reactive oxygen species is the major mediator of neuronal cell degeneration observed in neurodegenerative diseases, such as Alzheimer’s disease (AD) and vascular dementia (VaD). Additionally, hypertension has been shown to be a positive risk factor for VaD. Therefore, the objective of this study was to investigate the effects of *Lactobacillus plantarum* strain TWK10 (TWK10)-fermented soymilk on the protection of PC-12 cells in H_2_O_2_-, oxygen-glucose deprivation (OGD)- and deoxycorticosterone acetate (DOCA)-salt-induced rat models of VaD. Notably, the viabilities of H_2_O_2_-treated PC-12 cells and OGD model were significantly increased by treatment with TWK10-fermented soymilk ethanol extract (*p* < 0.05). In addition, oral administration of TWK10-fermented soymilk extract in DOCA-salt hypertension-induced VaD rats resulted in a significant decrease in blood pressure (*p* < 0.05), which was regulated by inhibiting ACE activity and promoting NO production, in addition to decreased escape latency and increased target crossing (*p* < 0.05). In conclusion, these results demonstrated that TWK10-fermented soymilk extract could improve learning and memory in DOCA-salt hypertension-induced VaD rats by acting as a blood pressure-lowering and neuroprotective agent.

## 1. Introduction

Soybean and its related products provide high-quality proteins, contain no lactose or cholesterol and are beneficial for lactose intolerance patients and vegetarians. They are also rich in glucoside isoflavones, such as daidzin and genistin [1]. Previous studies have shown that soymilk fermented with probiotics such as lactic acid bacteria (LAB), or its metabolites inhibit pathogen growth [2], blocks melanin production [3,4], suppresses serum cholesterol [5,6], exhibits anti-obesity effects [7] and modulates the immune system [2,8]. LAB-fermented soymilk also reduces the risk of atherosclerosis and related cardiovascular diseases [9,10].

Hypertension is a key symptom of metabolic syndrome, which is an important risk factor for the development of cardiovascular disease, myocardial infarction and stroke [11]. Previous studies have demonstrated that elevated blood pressure (BP) is strongly associated with the long-term risks of dementia and cognitive impairment [12]. As such, BP-lowering interventions may reduce the risk of cognitive impairment by their direct effects on cerebrovascular disease prevention or by indirect effects on the clinical incidence of neurodegenerative processes [13,14].

Sharma and Singh [15] (2012) demonstrated that treatment with deoxycorticosterone acetate (DOCA)-salt significantly raises the mean arterial BP of rats. These hypertensive rats performed poorly in subsequent Morris water maze (MWM) trials, indicative of learning and memory impairment. Moreover, DOCA-salt treatment was also shown to disrupt vascular endothelial function and various biochemical parameters. In our previous study, we demonstrated that *Lactobacillus plantarum* TWK10-fermented soymilk extract could effectively lower BP in hypertensive rats 8 h after oral administration [16]. In the current study, the antioxidant activity of TWK10-fermented soymilk and its protective effects on H_2_O_2_- and oxygen-glucose deprivation (OGD)-stimulated damage in PC-12 cells were determined. In addition, DOCA-salt-induced hypertension and associated dementia was monitored in rats following oral administration of TWK10-fermented soymilk to further characterise the potential protective effects.

## 2. Materials and Methods

### 2.1. Chemicals and Cell Culture

Lactobacilli de Man, Rogosa, and Sharpe (MRS) broth and Bacto agar were purchased from Becton, Dickinson and Company (Franklin Lakes, NJ, USA). Purified angiotensin-converting enzyme (ACE) from rabbit lung, captopril (CAP), hippuric acid, hippuryl-l-histidyl-l-leucine (HHL), *N*-[3-(2-furyl) acryloyl]-l-phenylalanylglycyl-glycine (FAPGG), l-ascorbic acid (AsA), 3-[4,5-dimethylthiazol-2-yl]-2,5-diphenyltetrazolium bromide (MTT) dye, DOCA, 2′,7’-dichloroflurescein diacetate (DCFH-DA) and dimethyl sulfoxide (DMSO) were purchased from Sigma (St. Louis, MO, USA). Non-genetically modified soybeans (*Glycine max* L. Merrill BB50) were obtained from ChuanGui Bio-Organic Co. (Taoyuan, Taiwan). The bacterial strain *L. plantarum* TWK10 was isolated from Taiwanese fermented cabbage and stored at −80 °C in Lactobacilli MRS with 20% glycerol [4]. PC-12 cells (BCRC60048) were obtained from the Bioresource Collection and Research Centre, Food Industry Research and Development Institute (Hsinchu, Taiwan) and cultured in RPMI-1640 medium containing 10% horse serum and 5% foetal bovine serum (HyClone Labs Inc., Thermo Fisher Scientific, Novato, CA, USA) at 37 °C in a humidified atmosphere containing 5% CO_2_. When confluent, cells were detached with 0.05% (w/v) trypsin/0.02% (w/v) ethylenediaminetetraacetic acid (EDTA) and resuspended in an appropriate medium for use in subsequent procedures. 

### 2.2. Preparation of Soymilk and Fermented Soymilk with TWK10 and Its Extracts

Soymilk was prepared according to the method described by Cheng *et al.* [17]. The soybeans were soaked in deionized water for 8 h at 25 °C. The swollen beans were ground into a homogenate using a food blender, with water equal to eight times (1:8) the dry weight of the soybeans and subsequently centrifuged with a sieve to obtain the supernatant, which was then heated in a water bath at 90 °C for 1 h. The culture strain was inoculated at 1% v/v to soymilk. The cultured soymilk samples were incubated in flasks at 37 °C for 48 h before being freeze dried (SDF-25 Freeze dryer; Chang Jung Business Co., Feng-Jen, Taiwan). The dry soymilk powder was extracted with water or 95% ethanol by shaking in a rotary shaker at 120 rpm and 25 °C for 2 h and then filtered through Waterman No. 42 filter paper. The filtrate was successively dried in vacuo. The dried materials were dissolved in water to provide water extract samples and the ethanol extract samples were dissolved in DMSO. The glucoside and aglycone isoflavones were analysed using high-performance liquid chromatography (HPLC) (Jasco Co., Tokyo, Japan), according to the method described by Kao and Chen [18].

### 2.3. Measurement of Superoxide Anion Radical Scavenging, Reducing Power and Ferrous Ion-Chelating Activities

The scavenging effects of extracts from TWK10-fermented soymilk on the α,α-diphenyl-β-picrylhydrazyl (DPPH) free radical were measured according to methods described by Yamaguchi with some modifications [19]. A volume of 100 μL of each sample was added to 500 μL of 0.1 mM DPPH in 95% ethanol. The mixture was shaken and left for 60 min at room temperature and the absorbance of the resulting solution was measured at 517 nm. In addition, the reducing power and ferrous ion-chelating activity of extracts from TWK10-fermented soymilk were measured as described by Moein *et al.* [20] and Dinis *et al.* [21], respectively.

### 2.4. Cell Culture, Viability Measurement, and Sample Treatment

PC-12 cells (8 × 10^3^ cells/well) seeded in a 96-well plate were treated with 100 μM H_2_O_2_ for 0.5–2 h to screen the 50% viable doses [22]. Cell viability was assessed using MTT assays [23]. Briefly, 8 × 10^3^ PC-12 cells per well were seeded in 24-well poly-L-lysine-coated microtiter plates, cultured for 24 h at 37 °C and transferred to differentiation medium containing nerve growth factor (NGF) for 6 days. Then, TWK10-fermented soymilk water extracts were added to cell cultures at a final concentration of 50, 100, 250, 500, or 1000 μg/mL and incubated for 24 h at 37 °C. Alternatively, TWK10-fermented soymilk ethanol extracts were added to cell cultures at a final concentration of 12.5, 25, 50, 100, or 200 μg/mL. Subsequently, 100 μM H_2_O_2_ was added, and samples were incubated for 30 min to evaluate the protective effects of TWK10-fermented soymilk against H_2_O_2_-induced cytotoxicity in PC-12 cells. In addition, OGD was performed according to method described by Singh *et al.* [24] with some modifications after treatment samples. The medium was replaced with pre-warmed RPMI-1640 medium without glucose. The cell cultures were then transferred into an anaerobic chamber equilibrated with 95% N_2_ and 5% CO_2_. The chamber was kept in a 37 °C incubator. After 6 h, cultures were placed back to the normoxic incubator with normal culture medium. The cell viability was measured by MTT assay [23]. Then, 500 μL of 5 mg/mL MTT diluted in phosphate-buffered saline (PBS; pH 7.4) was added to each well and plates were incubated at 37 °C for 1.5 h. The resulting formazan precipitate was solubilised by addition of 150 μL isopropyl alcohol containing 0.04 M hydrochloric acid. Absorbance was measured at 550 nm on a spectrophotometric microplate reader (Thermo Fisher, Inc., Waltham, MA, USA). Results were normalised such that the absorbance of solubilised precipitates from cells incubated without extract was set at 100%.

### 2.5. Measurement of Intracellular Reactive Oxygen Species (ROS) Generation

The measurement of intracellular ROS generation was performed according to method described by Ye *et al.* [16]. The cells were collected by pipetting and washed one time with PBS. After the addition of DCFH-DA (1 μM) to cell cultures and incubated for 1 h at 37 °C, the cells were washed twice with PBS. Ten thousand cells per sample were analysed by flow cytometry (Beckman Coulter, Inc., Brea, CA, USA) with the mean fluorescence intensity in the positive cells representing the amount of ROS present in the sample. 

### 2.6. Induction of Hypertension and Subsequent VaD in Rats

Hypertension and VaD were induced in rats by administering DOCA and a salt solution for 90 days as previously recorded [15]. A total of 30 Wistar rats (8-weeks-old, 260–300 g) were used in this experiment (BioLASCO Taiwan Co., Ltd., Taipei, Taiwan). Animals were maintained in the Fu Jen Laboratory Animal Centre (Taipei, Taiwan) at a temperature of 21 ± 2 °C and 55% ± 10% relative humidity with 12-h light/dark cycles. Rats were fed a standard diet (5010-Laboratory Rodent Diet; LabDiet, St. Louis, MO, USA) and given free access to tap water. DOCA was administered subcutaneously at 20 mg/kg twice weekly for 90 days. Primary exposure to the MWM occurred on day 85 and treatment continued during acquisition (days 86–89) and retrieval trials (day 90). Drinking water was then replaced with a solution of 1% NaCl and 0.2% KCl. Behavioural and other assessments were performed on day 86. All animal experiments were reviewed and approved by the Animal Care and Research Ethics Committee of the Fu Jen Catholic University (IACUC Approval No.: A10173).

### 2.7. Analysis of the Antihypertensive Effects of TWK10-Fermented Soymilk Extract over Long-Term Intake

TWK10-fermented soymilk water (Group DW) and ethanol (Group DE) extract samples were dissolved in distilled water (1 mL) and administered orally by gastric intubation between 12 and 1 p.m. Distilled water and the antihypertensive agent CAP (50 mg/kg) were used as negative (Group NC) and positive (Group CAP) controls respectively [25]. The dose of TWK10-fermented soymilk extract was based on 450 mL/day/person (weight, 65 kg; height, 170 cm), which was converted to animal equivalent doses using the body surface area formula [26]. The 1× dose of TWK10-fermented soymilk water or ethanol extracts powder for rats were 2.65 and 0.09 g/kg body weight, respectively.

Systolic BP (SBP) and diastolic BP (DBP) were recorded using the tail-cuff method before administration and post administration at 2nd, 5th, 7th, 9th and 11th week, using a BP-2000 Blood Pressure Analysis System (Visitech System, Inc., Apex, NC, USA). Rats were kept at 37 °C for 40 min to detect pulsations in the tail artery prior to measurement. Five readings were taken and the mean of all measurements was calculated. To minimise stress-induced variations in BP, the same person made all measurements in the same calm environment. A training period of 2 weeks was established before the actual trial time, during which time the rats became accustomed to the procedure, to guarantee measurement validity.

### 2.8. Assessment of Learning and Memory Using the Interoceptive Behaviour Model

A modified MWM task was used to evaluate memory and learning ability from day 84 to day 90 [27,28]. A black circular tank (diameter, 160 cm; height, 60 cm) was used as the apparatus of the MWM in which a movable escape platform (diameter, 10 cm; height, 25 cm) was located inside the tank. The tank was filled to a height of 27.5 cm with water (temperature: approximately 23 °C) and the surface of the platform was 2.5 cm below the surface of the water. The tank was divided into four quadrants and a position with equal distance from the centre and edge, in the middle of each quadrant, was marked for the location of the platform. The tank was located in a test room with many cues external to the maze. The room had adjustable indirect light and a camera was set at the ceiling above the centre of the water tank. The position of the cues remained unchanged throughout the water maze task.

The day before the experiment was dedicated to swim training for 60 s in the absence of the platform and the reference memory task was performed on the following days. Starting positions were randomised daily and quadrant 4 (Q4) was maintained as the target quadrant in all acquisition trials. Each animal was subjected to four consecutive trials per day with a gap of 1.5 min. The rats were gently placed in the water between quadrants facing the wall of the pool and given 90 s to locate the submerged platform. The rats were then allowed to stay on the platform for 30 s. Rats that failed to find the platform within 90 s were gently guided onto the platform and allowed to remain there for 30 s. The mean time and swimming distance spent in all four quadrants were recorded and the time and swimming distance spent in the target platform were used to provide an index of retrieval. The day after the last trial session, animals were subjected to a probe trial session in which the platform was removed from the pool and rats were then allowed to swim for 90 s to search for the platform. A record was kept of the swimming time, swimming pathway and the number of animals that swam to the area within the quadrant, where the platform had been previously placed.

A five-trial working memory task was performed from day 88 to day 90, as previously described, with minor modifications [29,30]. The rules and apparatus for the working memory task were the same as those for the reference memory task, except that the platform was located in a new quadrant each day. The rat was placed into the water tank at one of the five different starting positions in each trial. The first trial of each session per day was recorded as an informative practice trial where the rat was allowed to remain on the platform for 15 s and then returned to its home cage in order to rest. The next trial started after a rest period of 60 s. The platform was located in the same position and the trials were repeated a total of five times. The working memory task was designated as the mean escape latency from trials 2–5 and was assessed for each rat on 3 consecutive days.

### 2.9. Biochemical and Histological Assessment

All animals were fasted for 24 h before sacrifice by carbon dioxide inhalation. Blood was collected by cardiac puncture and serum samples were obtained by drawing the blood into a serum separated tube, allowing it to clot and then centrifuging for 10 min at 3000× *g* to separate serum. Plasma was obtained by collecting blood in heparinised syringes containing 5% heparin and 2% sodium citrate and centrifuging at 3000× *g* for 15 min. The aorta, liver, lung and kidney tissues were harvested and divided into two parts. One part was homogenised (0.1 g in 1 mL PBS) using a FastPrep System (MP Biomedicals, Santa Ana, CA, USA) and the other was immediately fixed in 10% neutral buffered formalin for further histological analysis.

ACE activity in lung and kidney tissue was determined, as described by Cushman and Cheung with some modification [31]. Aliquots (50 μL) of buffered substrate solution (12.5 mM HHL in 100 mM borate buffer solution containing 300 mM NaCl; pH 8.3) were mixed with samples (50 μL) and then pre-incubated at 37 °C for 5 min. ACE (25 μL; 25 mU/mL) was added to the reaction, which was incubated at 37 °C for 20 min. The enzymatic reaction was stopped by adding 0.5 N HCl (1 mL). The hippuric acid released because of ACE activity was extracted with ethyl acetate (1 mL), dried and resuspended in deionised water (1 mL), and the absorbance at 228 nm was measured. Inhibitory activity was calculated using the following equation: ACE inhibitory activity (%) = 100 × ([A − B] − [C − D])/(A − B), where A is the absorbance of a solution containing ACE but no sample, B is the absorbance of a solution containing ACE that had been previously inactivated by the addition of HCl but no sample, C is the absorbance of a solution containing both ACE and the sample and D is the absorbance of a solution containing HCl-inactivated ACE and the sample.

Ang II serum levels were measured using an Ang II EIA kit (Phoenix Pharmaceuticals, Inc., Burlingame, CA, USA). The levels of nitrite (NO^2−^) and nitrate (NO^3−^) anions in plasma were measured using a nitric oxide EIA kit (Cayman Chemical Company, Ann Arbor, MI, USA). Serum lipid peroxide was determined by measuring the thiobarbituric acid reactive substance (TBARS) concentration as an index of lipid peroxidation, as previously described [32]. Erythrocyte catalase (CAT) activity in whole blood was determined as previously described [33]. In addition, SOD and acetylcholinesterase activity and glutathione levels were measured using commercial kits (SD125 from Randox Laboratories Ltd., Crumlin, UK; ab138871 from Abcam, Cambridge, MA, USA and No. 703002 kit from Cayman Chemical Company).

Vascular elastin distribution and nitric oxide synthase (eNOS) protein expression were evaluated in aorta samples by optical microscopy. For this, the tissue was embedded in paraffin and sections were stained with hematoxylin-eosin. Verhoeff’s stain was used to evaluate vascular elastin expression and distribution [34]. The microstructural changes in the vascular elastin were assessed by determining the number of elastin bands in several sections of each aorta according to Hussein *et al.* [15]. eNOS protein expression was quantified with an immunohistochemical assay according to the method of Zhao *et al.* [35] and examined using a Nikon TS-100 microscope (Tokyo, Japan). Motic Images 2000 software (Xiamen, China) was used to examine changes within the hypertensive tissues.

### 2.10. Statistical Analysis

All values represent the means and standard deviations of three independent experiments. Data were compared using Duncan’s multiple range method with SPSS statistical analysis software (IBM Software, Armonk, NY, USA).

## 3. Results

### 3.1. Effects of TWK10-Fermented Soymilk on Antioxidant Activities

Table 1 shows the effects of extracts from TWK10-fermented soymilk on DPPH scavenging activity, ferrous ion chelation and total reducing power. EDTA (0.5 µg/mL) and ASA (1 mg/mL) were used as positive controls for the tests of ferrous ion chelation and total reducing power, respectively. The results showed that EDTA had ferrous chelating effects (63.03% ± 8.91%) and ASA increased the total reducing power to 1.51 ± 0.14, indicating that the tests had been performed correctly in this study. There was no effect of unfermented and TWK10fermented soymilk water extracts on DPPH scavenging, in addition, unfermented soymilk water extracts also had no effect on ability for chelating Fe^2+^. The DPPH scavenging activity was increased by treatment with the ethanol extract of the unfermented soymilk, beginning at a concentration of 5 mg/mL. For the TWK10-fermented soymilk, the DPPH scavenging activity of the ethanol extract increased significantly with the sample concentration; in addition, a concentration-dependent effect was observed (*p* < 0.05). The DPPH scavenging activity of the ethanol extracts from TWK10-fermented soymilk was significantly higher than that from unfermented soymilk by 29.82% (*p* < 0.05) at a concentration of 20 mg/mL. The ethanol extract from unfermented soymilk exhibited ferrous chelating effects when compared with that from TWK10-fermented soymilk. Notably, both the water and ethanol TWK10-fermented soymilk extract exhibited concentration-dependent ferrous ion chelating effects (*p* < 0.05), of which the ethanol extract was more effective. In addition, the half-maximal inhibitory concentrations (IC_50_) of the water and ethanol extracts from TWK10-fermented soymilk were 4.28 and 4.08 mg/mL respectively, which were significantly higher than that of the ethanol extracts from unfermented soymilk (8.59 mg/mL). Furthermore, the extracts from unfermented or TWK10-fermented soymilk tended to increase the total reducing power as the sample concentration increased. In addition, the total reducing power of the TWK10-fermented soymilk was superior to that of the unfermented soymilk at a concentration of 20 mg/mL and that of the ethanol extract was superior to that of the water extract (*p* < 0.05). Thus, TWK10 soymilk fermentation significantly improved its total reducing power.

### 3.2. Protective Effects of TWK10-Fermented Soymilk Extracts on PC-12 Cell Viability Subjected to H_2_O_2_-Induced Oxidative Stress and OGD

**Figure 1:**
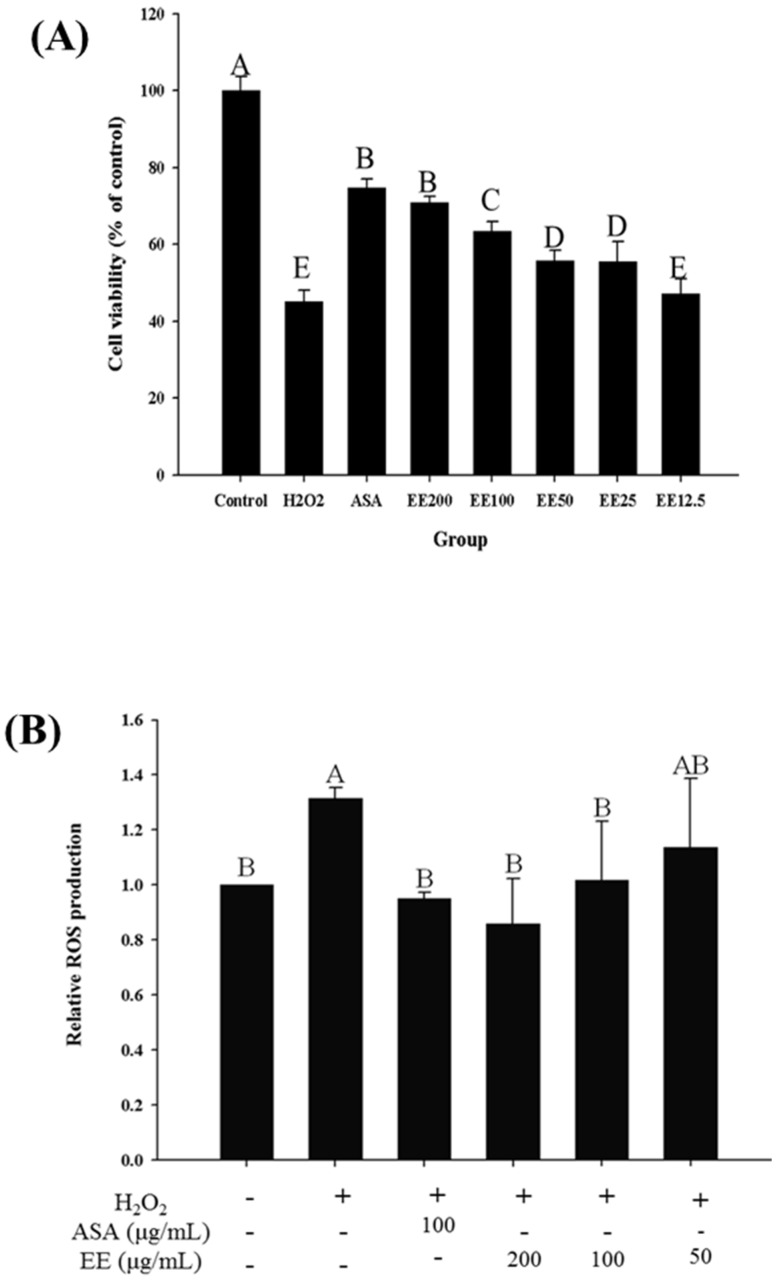
Effect of the ethanol extract of TWK10-fermented soymilk on cell viability (**A**) and ROS production (**B**) of an H_2_O_2_ treatment oxidative stress model in PC-12 cells; Data are presented as means ± SD (*n* = 3); Bar values bearing different letters were significantly different in accordance by Duncan’s multiple range test (*p* < 0.05); ASA: ascorbic acid (100 μg/mL); H_2_O_2_: dihydrogen dioxide (100 μM); EE200: 200 μg/mL of ethanol extract of TWK10-fermented soymilk; EE100: 100 μg/mL of ethanol extract of TWK10-fermented soymilk; EE50: 50 μg/mL of ethanol extract of TWK10-fermented soymilk; EE25: 25 μg/mL of ethanol extract of TWK10-fermented soymilk; EE12.5: 12.5 μg/mL of ethanol extract of TWK10-fermented soymilk.

The viability of PC-12 cells treated with H_2_O_2_ (100 µM) decreased significantly by 54.98% when compared with that of the control group (*p* < 0.05, Figure 1). In this study, 50% cell viability was used as the damage baseline, which further decreased to 50.50% ± 0.56% by treatment with H_2_O_2_ for 30 min. This treatment condition was used in subsequent studies. The protective effects of extracts from TWK10-fermented soymilk on H_2_O_2_-induced PC-12 cells were then analysed. ASA, which was used as the positive control, increased cell viability to 74.46% ± 2.19% (*p* < 0.05). Treatment with TWK10-fermented soymilk water extract at concentrations of 500 and 1000 µg/mL increased cell viability slightly to 51.38% ± 3.14% and 51.18% ± 5.14%, respectively (data not shown). Nevertheless, cell viability was significantly increased from 55.56% ± 5.17% to 70.99% ± 1.55% following treatment with the TWK10-fermented soymilk ethanol extract at concentrations ranging from 25 to 200 µg/mL (*p* < 0.05). In addition, no significant differences were observed between the experimental group at a concentration of 200 µg/mL and the positive control groups (*p* > 0.05). Furthermore, ethanol extract treatment significantly suppressed ROS production by 0.86–1.02 fold at concentrations from 50 to 200 µg/mL (*p* < 0.05).

**Figure 2:**
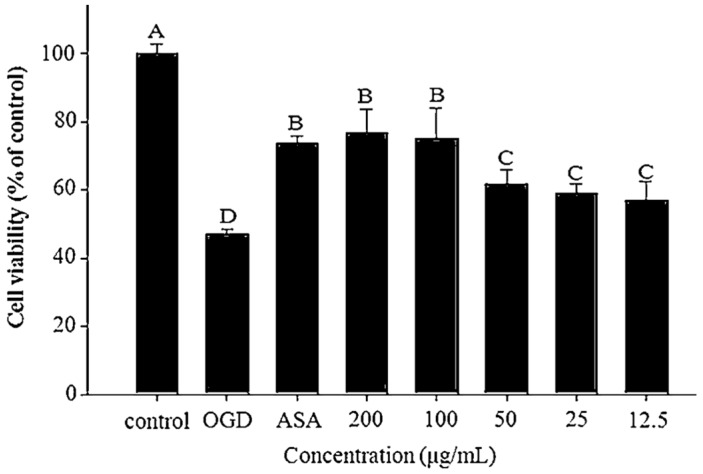
Effect of the ethanol extract of TWK10-fermented soymilk on cell viability of oxygen-glucose deprivation (OGD) model in PC-12 cells; ata are presented as means ± SD (*n* = 3); Bar values bearing different letters were significantly different in accordance by Duncan’s multiple range test (*p* < 0.05); ASA: ascorbic acid (100 μg/mL).

In the current study, OGD model was established using CO_2_ instead of O_2_ in an incubation chamber, demonstrating that PC-12 cells could survive under hypoxia. After a 6-h incubation, the viability of the PC-12 cells in the OGD model was reduced by 50% when compared with that of the control group (*p* < 0.05). Treatment with 100 µg/mL ASA as the positive control significantly increased cell viability by 73.70% ± 1.90% (*p* < 0.05, Figure 2). Moreover, TWK10-fermented soymilk water extract increased cell viability by 52.64% ± 1.44% to 57.02% ± 2.90% at concentrations ranging from 100 to 1000 µg/mL (*p* < 0.05), whereas the ethanol extract increased the cell viability by 57.06% ± 5.16% to 76.62% ± 7.00% at concentrations ranging from 12.5 to 200 µg/mL (*p* < 0.05). No significant differences were observed between the experimental and positive control groups at 200 µg/mL (*p* > 0.05).

### 3.3. Inhibitory Effects of TWK10-Fermented Soymilk on DOCA-Salt-Induced Hypertension

Rats subjected to 13 weeks of DOCA-salt-induced hypertension drank 1.88- to 5.14-fold more water than rats in the other groups (*p* < 0.05), consuming 108.09–185.95 mL water per day. No significant differences in food intake were observed among the various groups. In addition, the body weights of the rats slowly increased following DOCA-salt-induced hypertension, supporting that the model was correctly established.

Table 2 shows the results of SBP measurements in the rats. No significant differences in SBP were observed among the various groups before sample administration (0 weeks), indicating that the BPs of these rats were the same at baseline. Following DOCA-salt-induced hypertension, SBPs in the D, CAP, DW and DE groups increased significantly from 32.03 to 34.40 mmHg after 7 weeks of induction when compared with those in negative controls (*p* < 0.05). Conversely, the SBP measurements in rats administrated TWK10-fermented soymilk water and ethanol extracts for 2 weeks were 4.94% and 6.79% lower than those in group D respectively (*p* < 0.05). Moreover, the SBP measurements in rats administrated TWK10-fermented soymilk water and ethanol extracts for 4 weeks were suppressed by 11.23% and 14.50% (*p* < 0.05) respectively.

### 3.4. Effects of TWK10-Fermented Soymilk Extracts on DOCA-Salt-Induced Hypertension and Associated Dementia (Morris Water Maze)

In the current study, the learning ability and memory of rats with DOCA-salt hypertension was evaluated using a Morris water maze. After 3 days of training, both the swimming distance and time required for rats to reach the escape platform decreased significantly (*p* < 0.05), indicating that all groups demonstrated learning abilities (Table 3). As hypothesised, the swimming distance and escape latency for group D to reach the escape platform were significantly longer than those of the other groups following initiation of DOCA-salt-induced hypertension (*p* < 0.05), indicating that the feeding of DOCA-salt had influenced their learning ability. On day 3, compared with group D, the swimming distances required for groups DW and DE to reach the escape platform were significantly reduced by 485.07 cm (61.02%; *p* < 0.05) and 612.05 cm (76.99%; *p* < 0.05), respectively. In addition, the escape latency for groups DW and DE were reduced by 17.47 s (64.46%) and 21.35 s (78.78%) respectively, when compared with that of group D (*p* < 0.05). Thus, feeding TWK10-fermented soymilk extract to rats had improved their learning ability.

**Figure 3:**
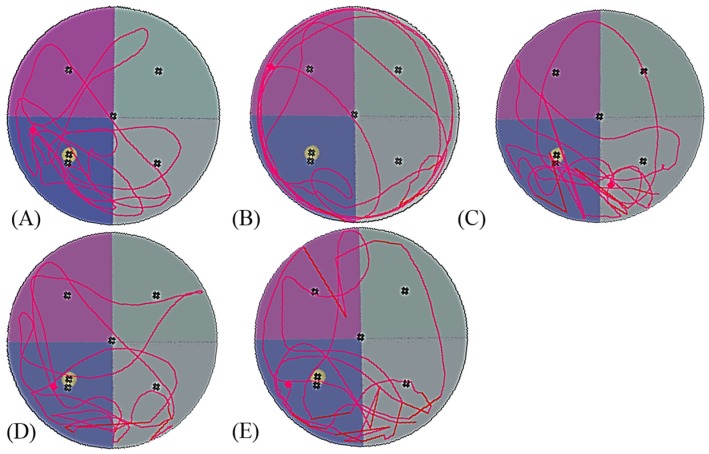
Effects of administration of the extracts from TWK10-fermented soymilk on swimming pathways in Morris water maze in DOCA-salt hypertension induced vascular dementia rats NC (**A**); D (**B**); CAP (**C**); DW (**D**); and DE (**E**). NC: normal control group; D: DOCA-salt at a dose of 20 mg/kg bw; CAP: DOCA-salt with administration of captopril at a dose of 50 mg/kg bw; DW: DOCA-salt with administration of water extract of TWK10-fermented soymilk at a dose of 2.65 g/kg bw; DE: DOCA-salt with administration of ethanol extract of TWK10-fermented soymilk at a dose of 0.09 g/kg bw.

A probe trial session was used to assess the long-term memories of the rats. As shown in Figure 3, the frequency of crossing the escape platform was higher in groups NC, CAP, DW, and DE than that in group D. Additionally, the recorded swimming paths for group D were disorganised when compared with those of other groups, indicating memory deterioration. Quantitative results showed that compared with groups without DOCA-salt-induced hypertension, the frequency of crossing the escape platform for the group D rats was significantly reduced to 1.33 ± 1.21 (*p* < 0.05; Table 4). The frequencies for rats in the groups treated with the DW and DE samples also increased to 2.83 ± 1.33 and 3.33 ± 1.03 (*p* < 0.05), respectively. No significant differences were observed in the frequencies of the DW and DE groups and negative controls (*p* > 0.05). The duration that rats in groups NC, CAP, DW and DE stayed in nontarget quadrants were reduced substantially. Therefore, administration of TWK10-fermented soymilk extracts significantly enhanced the spatial and long-term memory in the hypertensive rats (*p* < 0.05).

A working memory test was conducted to evaluate short-term memory. Notably, the swimming distance required for group D rats with DOCA-salt-induced hypertension to reach the escape platform was significantly increased by 441.56 cm (*p* < 0.05; Table 5); however, after administration of TWK10-fermented soymilk extracts, the swimming distances for rats in groups DW and DE were reduced by 453.40 and 466.61 cm (*p* < 0.05), respectively. When compared with group D, the escape latencies for groups DW and DE were reduced by 17.87 ± 15.63 s and 18.07 ± 15.70 s (*p* < 0.05) respectively, indicating that the TWK10-fermented soymilk extracts improved the rats’ short-term memory.

### 3.5. Effects of TWK10-Fermented Soymilk Extracts on the Activities of SOD, AChE and CAT and the MDA and GSH Contents in the Brains of Rats with DOCA-Salt-Induced Hypertension

Table 6 shows the SOD activities and MDA contents in the brain tissues of rats. Compared with the control group, the SOD activities in the brain tissues of rats in group D were significantly reduced by 67.22% (*p* < 0.05), indicating that antioxidant activity was reduced in rats after DOCA-salt induction. After administration of TWK10-fermented soymilk extracts for 5 weeks, the SOD levels in the brain tissues of rats in groups DW and DE were significantly increased by 0.83 and 2.15 times, respectively. In addition, free radicals increased following induction so lipid peroxidation increased and the MDA levels in rats became considerably higher than those in the control group (*p* < 0.05). Administration of TWK10-fermented soymilk extracts effectively suppressed MDA serum levels from 26.63% to 38.08% (*p* < 0.05), indicating that TWK10-fermented soymilk extracts could limit oxidative stress.

Interestingly, brain AChE activity in DOCA-salt-induced hypertensive rats was significantly increased when compared with that in the control group, whereas CAT activity and GSH levels decreased. These data indicated that oxidative stress in the rats’ brains had increased (*p* < 0.05). As expected, administration of TWK10-fermented soymilk extracts for 5 weeks reduced AChE activities in the brains of rats in groups DW and DE by 46.49 and 36.24 mU/mL, respectively (*p* < 0.05), while CAT activities were increased by 125.78 and 124.82 mU/mL, respectively, and GSH levels were increased by 7.76 and 14.24 µM, respectively.

### 3.6. Regulatory Effects of TWK10-Fermented Soymilk Extract Administration on DOCA-Salt-Induced Hypertension and Associated Dementia

Serum Ang II levels in rats in group D were significantly higher than those in rats in group NC (Table 7; *p* < 0.05) indicating that this was a key parameter in DOCA-salt-induced hypertension. Notably, Ang II levels were significantly suppressed following administration of TWK10-fermented soymilk extracts (*p* < 0.05), consistent with the observations for SBP.

**Figure 4:**
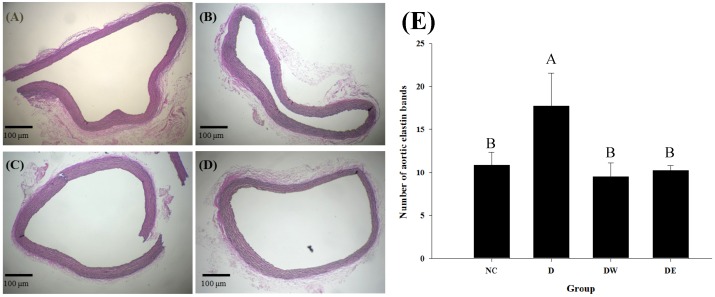
The effect of the extracts of TWK10-fermented soymilk on microscopic examination of aorta elastin bands number in DOCA-salt hypertension induced vascular dementia rats NC (**A**); D (**B**); DW (**C**); DE (**D**) and number (**E**). Data are presented as mean ± SD (*n* = 3); NC: Normal control group; D: Subcutaneous administration of DOCA-salt at a dose of 20 mg/kg bw; DW: DOCA-salt with administration of water extract of TWK10-fermented soymilk at a dose of 2.65 g/kg bw; DE: DOCA-salt with administration of ethanol extract of TWK10-fermented soymilk at a dose of 0.09 g/kg bw.

Figure 4 shows microscopic images of Verhoeff-stained aortic arches, which were used to evaluate elastin distribution. Importantly, the disorganised elastin arrangement apparent in DOCA-salt-induced hypertensive rats was somewhat ameliorated following administration of TWK10-fermented soymilk extracts (groups DW and DE *vs.* group D). Furthermore, the amount of elastin bands in the aortic arches of rats in group D was 17.75 ± 3.83 bands, which was significantly higher than that in rats in group NC (*p* < 0.05). Moreover, the amount of elastin was significantly lower than that in the control group after treatment (*p* < 0.05), indicating that feeding TWK10-fermented soymilk extracts to hypertensive rats reduced their BP and increased the likelihood of elastin restructuring.

**Figure 5:**
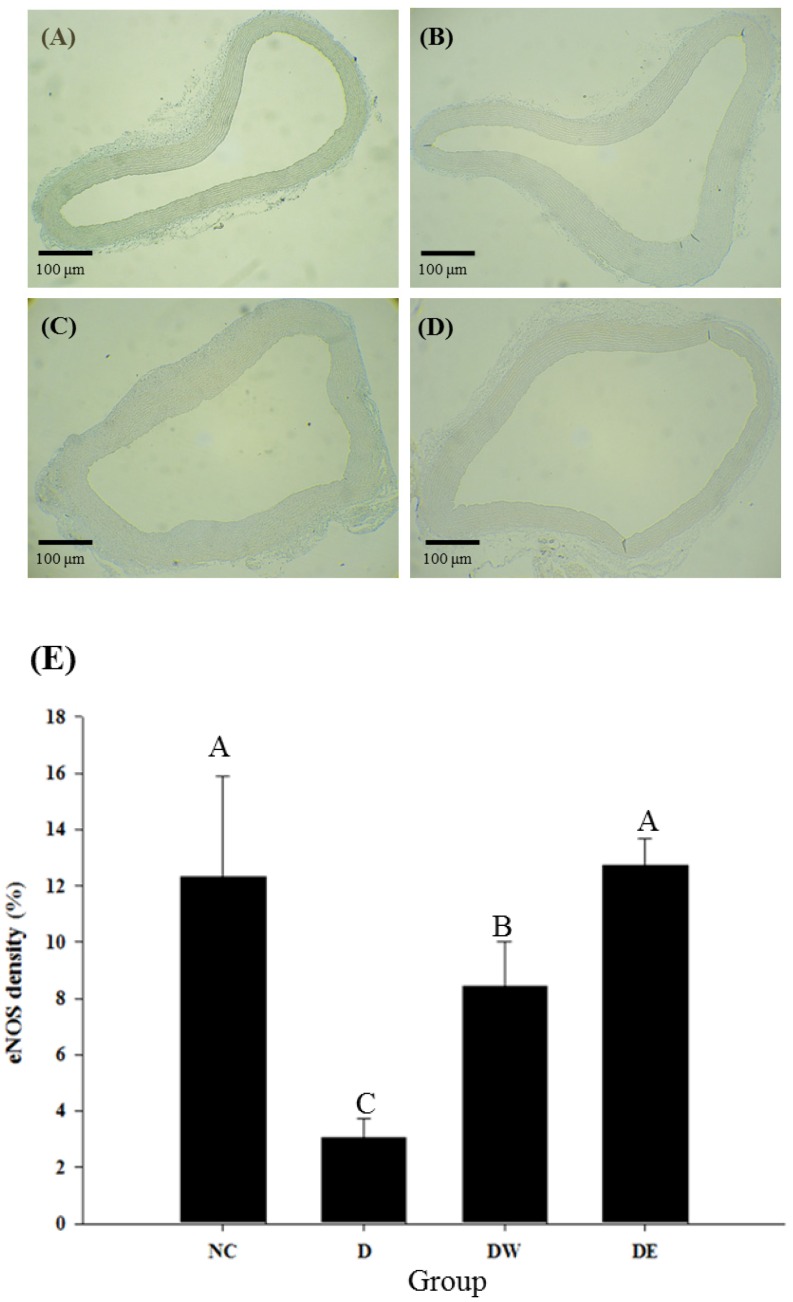
Effects of administration of the extracts of TWK10-fermented soymilk on immunohistochemical of eNOS in the sections of aorta in DOCA-salt induced hypertension and its associated dementia rats NC (**A**); D (**B**); DW (**C**); DE (**D**); and eNOS density (**E**). (100 magnification); Data are presented as means ± SD (*n* = 6); Bar values with different uppercase letters were significantly different by Duncan’s multiple range test (*p* < 0.05); NC: normal control group; D: DOCA-salt at a dose of 20 mg/kg bw; DW: DOCA-salt with administration of water extract of TWK10-fermented soymilk at a dose of 2.65 g/kg bw; DE: DOCA-salt with administration of ethanol extract of TWK10-fermented soymilk at a dose of 0.09 g/kg bw.

Table 7 shows the effects of TWK10-fermented soymilk extracts on plasma Nitrate/nitrite levels and lung ACE activity in DOCA-salt-induced hypertensive rats. Most notably, plasma Nitrate/nitrite levels increased 24.15% and 33.46% following treatment with TWK10-fermented soymilk water and ethanol extracts, respectively, when compared with that of the D group (*p* < 0.05). Interestingly, Nitrate/nitrite production was significantly higher in rats in group DE than in rats in group DW (*p* < 0.05). Immunostaining confirmed that administration of water and ethanol extracts significantly increased eNOS expression within arcuate arteries by 2.75- and 4.16-fold respectively, compared with that observed in the control group (*p* < 0.05; Figure 5). In addition, eNOS expression was significantly higher in DE rats than in DW rats (*p* > 0.05), consistent with the results of Nitrate/nitrite levels in treated DOCA-salt-induced hypertension rats. Moreover, water and ethanol extracts were found to significantly suppress ACE activity in the lungs of treated rats (*p* < 0.05). 

## 4. Discussion

Studies have indicated that β-glucosidase in lactic acid bacteria catalyses the release aglycone isoflavones via glucoside isoflavone hydrolysis during the fermentation process [36,37]. Aglycone isoflavones possess antioxidative properties that can clear free radicals and chelate metal ions [38,39,40]. Additionally, Chen *et al.* [4] and Cheng *et al.* [17] indicated that the content of aglycone isoflavones was significantly higher in TWK10-fermented soymilk than in soymilk fermented with other strains; therefore the bioactive content in the soymilk was also higher. TWK10-fermented soymilk ethanol extract contains 75.71 ± 7.92 and 144.34 ± 2.29 µg/mL genistein and daidzein, respectively; therefore the antioxidative capabilities of ethanol extracts are correlated with water extract material and aglycone isoflavone content. In the current study, our *in vitro* antioxidant evaluation revealed that TWK10-fermentation significantly increased antioxidant activities and that the anti-oxidative capability of the ethanol extract was superior to that of the water extract, likely due to the activity of aglycone isoflavones.

Hypertension increases the risks of endothelial dysfunction and VaD [41]. Endothelial dysfunction is associated with many diseases and conditions such as atherosclerosis, chronic heart failure, chronic renal failure, hypertension, stroke and dementia. Notably, improving endothelial dysfunction can not only help VaD but also reduce symptoms of late-onset AD [42,43,44]. Thus, hypertension is a crucial indicator of these diseases. Complete and proper functioning of the vascular system is critical for efficient brain function. For example, high BP can cause cerebral vascular endothelial and smooth muscle cell rupture, leading to vascular dysfunction [45,46]. Dai [47] noted that excessive generation of peroxide which caused imbalance, could affect synaptic plasticity, nerve signalling and memory impairment; therefore, effectively reducing ROS content and enhancing antioxidative capacity can limit the nerve cell damage caused by oxidative stress.

The regulation of BP is primarily associated with the renin-angiotensin-aldosterone system (RAAS). Effectively lowering the activities of key enzymes in the RAAS, such as ACE and renin, can further reduce nicotinamide adenine dinucleotide phosphate oxidase (NOX) activity and ROS content, as well as increase antioxidant capability [48]. Thus, cognitive deficits caused by hypertension and the associated dementia in DOCA-salt-induced rats can be improved [49,50]. In this study, rats that were continually fed TWK10-fermented soymilk extract exhibited reduced Ang II content, increased NO production and a normalised BP. In addition, the antioxidant content in the brain was normalised and hypertension was reduced.

Although the effect of the water extract was inferior to that of the ethanol extract in the *in vitro* antioxidant test, uracil and glycerol were identified as the functional ingredients in the TWK10-fermented soymilk water extract as determined by their NO-promoting role in human umbilical vein endothelial cell (HUVEC) generation identified in our previous study, indicating that this combination could regulate BP, primarily because of the mixing ratio of uracil and glycerol. After mixing, these two ingredients exhibited additive properties and could effectively lower BP [16]. Furthermore, studies have shown that oxidative stress along with increased Ang II content is one of the main causes of high BP [51]. In the DOCA-salt model, in addition to increasing Ang II, large quantities of free radicals and oxidative stress were generated and induced an increase in BP [52]; genistein could lower oxidative stress and protect nerve cells [42]. Taken together, these data demonstrated that TWK10-fermented soymilk extracts could reduce the occurrence of dementia in DOCA-salt-induced rats through BP and oxidative stress regulation.

In conclusion, TWK10-fermented soymilk extracts exhibited antioxidant activities *in vitro* and protective effects on PC-12 cell viability subjected to H_2_O_2_-induced oxidative stress and OGD. Additionally, the water and ethanol extracts from TWK10-fermented soymilk could decrease BP by inhibiting ACE activity and promoting NO production. The water and ethanol extracts from TWK10-fermented soymilk were as effective as an existing drug, CAP, at lowering BP in DOCA-salt hypertension-induced VaD rats. Taken together, the water and ethanol extracts from TWK10-fermented soymilk also improved the learning ability of rats and reduced the occurrence of dementia through regulation of BP and oxidative stress in DOCA-salt hypertension-induced VaD rats. The purification and absorption of these functional or bioactive ingredients in water and ethanol extracts are interesting topics for future studies. Additionally, these data may be useful for developing functional foods, thereby extending the range of applications of crops containing these bioactive components.

## Figures and Tables

**Table 1:** The scavenging effect of DPPH radicals, ability for chelating Fe^2+^ and reducing activity of extracts of unfermented soymilk and TWK10-fermented soymilk.

Concentration (mg/mL)	DPPH Scavenging Effect (%)		Ability for Chelating Fe^2+^ (%)			Reducing Activity (OD_700_)			
	UN EE	TWK 10 EE	UN EE	TWK10 EE	TWK10 WE	UN EE	UN WE	TWK10 EE	TWK10 WE
20	47.40 ± 1.95 ^Ab^	77.22 ± 0.46 ^Aa^	54.22 ± 4.60 ^Ab^	70.78 ± 1.39 ^Aa^	55.32 ± 1.45 ^Ab^	0.762 ± 0.11 ^Ab^	0.530 ± 0.01 ^Ac^	0.959 ± 0.00 ^Aa^	0.607 ± 0.01 ^Ac^
10	40.86 ± 4.92 ^Bb^	75.46 ± 2.86 ^Aa^	55.30 ± 7.17 ^Ab^	61.84 ± 2.02 ^ABab^	53.94 ± 0.07 ^Ab^	0.526 ± 0.06 ^Bb^	0.385 ± 0.01 ^Bc^	0.663 ± 0.01 ^Ba^	0.414 ± 0.03 ^Bbc^
5	26.86 ± 1.47 ^Cb^	67.12 ± 5.21 ^Aa^	36.52 ± 8.73 ^Bc^	50.67 ± 6.12 ^Bbc^	54.04 ± 1.01 ^Ab^	0.407 ± 0.06 ^Bab^	0.307 ± 0.00 ^Cc^	0.471 ± 0.02 ^Ba^	0.340 ± 0.00 ^Bbc^
1	ND	26.69 ± 3.74 ^Ba^	10.73 ± 1.98 ^Cc^	28.06 ± 2.45 ^Cb^	31.49 ± 5.51 ^Bb^	0.250 ± 0.01 ^Cb^	0.226 ± 0.00 ^Db^	0.355 ± 0.10 ^BCa^	0.246 ± 0.00 ^Cb^
0.1	ND	17.53 ± 7.96 ^Ba^	6.07 ± 0.80 ^Cb^	11.32 ± 3.68 ^Dab^	16.15 ± 7.56 ^Cab^	0.211 ± 0.01 ^Cb^	0.210 ± 0.00 ^Db^	0.246 ± 0.02 ^Ca^	0.237 ± 0.00 ^Cab^

Data are presented as means ± SD (*n* = 3); Values with different uppercase letters were significantly different in column and values with different lowercase letters were significantly different in row of same test by Duncan’s multiple range test (*p* < 0.05); ND: non-detected; UN EE: ethanol extract of unfermented soymilk; UN WE: water extract of unfermented soymilk; TWK10 EE: ethanol extract of soymilk fermented with *Lactobacillus plantarum* TWK10; TWK10 WE: water extract of soymilk fermented with *L. plantarum* TWK10.

**Table 2:** Effects of administration of the extracts from TWK10-fermented soymilk on systolic blood pressure in DOCA-salt induced hypertension and its associated dementia rats.

Group	SBP (mmHg)					
	0-week	2-week	5-week	7-week	9-week	11-week
NC	127.50 ± 3.83 ^Aa^	126.28 ± 1.26 ^Ba^	126.44 ± 2.24 ^Ca^	127.67 ± 1.74 ^Ba^	128.21 ± 3.25 ^Da^	126.82 ± 2.82 ^Da^
D	127.79 ± 3.25 ^Ae^	139.12 ± 1.92 ^Ad^	152.08 ± 5.15 ^Bc^	159.79 ± 6.93 ^Ab^	162.92 ± 7.65 ^Aab^	167.86 ± 4.43 ^Aa^
CAP	127.23 ± 3.16 ^Ad^	139.64 ± 4.21 ^Ac^	150.00 ± 6.24 ^Bb^	161.39 ± 4.22 ^Aa^	143.95 ± 7.07 ^Cbc^	140.26 ± 8.70 ^Cc^
DW	126.57 ± 2.42 ^Ad^	138.69 ± 2.32 ^Ac^	160.02 ± 8.40 ^Aa^	159.69 ± 4.53 ^Aa^	154.87 ± 4.12 ^Ba^	149.00 ± 6.68 ^Bb^
DE	125.95 ± 2.43 ^Ad^	139.03 ± 3.28 ^Ac^	152.22 ± 5.51 ^Bb^	162.07 ± 4.91 ^Aa^	151.85 ± 3.23 ^Bb^	143.51 ± 9.11 ^BCc^

Data are presented as means ± SD (*n* = 6); Values with different uppercase and lowercase letters in the same column and in the same row were significantly different respectively, by Duncan’s multiple range test (*p* < 0.05); SBP: systolic blood pressure; NC: normal control group; D: DOCA-salt at a dose of 20 mg/kg bw; CAP: DOCA-salt with administration of captopril at a dose of 50 mg/kg bw; DW: DOCA-salt with administration of water extract of TWK10-fermented soymilk at a dose of 2.65 g/kg bw; DE: DOCA-salt with administration of ethanol extract of TWK10-fermented soymilk at a dose of 0.09 g/kg bw.

**Table 3:** Effects of administration of the extracts from TWK10-fermented soymilk on reference memory task in Morris water maze in DOCA-salt induced hypertension and its associated dementia rats.

Group	Total Swimming Distance (cm)			Escapes Latency (s)		
	Day 1	Day 2	Day 3	Day 1	Day 2	Day 3
NC	440.22 ± 96.73 ^Ca^	400.85 ± 95.60 ^Ba^	312.33 ± 130.41 ^Ba^	13.81 ± 5.64 ^Ca^	10.51 ± 4.23 ^Ba^	8.67 ± 4.08 ^Ba^
D	1743.62 ± 633.50 ^Aa^	1275.05 ± 552.65 ^Aab^	794.95 ± 237.93 ^Ac^	72.79 ± 16.63 ^Aa^	46.30 ± 24.73 ^Ab^	27.10 ± 10.52 ^Ab^
CAP	983.41 ± 439.07 ^Ba^	478.58 ± 123.01 ^Bb^	308.44 ± 127.76 ^Bb^	40.45 ± 29.25 ^Ba^	13.72 ± 8.00 ^Bb^	10.47 ± 5.32 ^Bb^
DW	885.23 ± 275.23 ^BCa^	410.71 ± 189.39 ^Bb^	309.88 ± 112.72 ^Bb^	35.27 ± 13.20 ^BCa^	17.35 ± 6.45 ^Bb^	9.63 ± 3.83 ^Bb^
DE	701.34 ± 215.24 ^BCa^	358.15 ± 157.46 ^Bb^	182.90 ± 76.68 ^Bb^	25.75 ± 14.00 ^BCa^	10.01 ± 4.30 ^Bb^	5.75 ± 3.35 ^Bb^

Data is presented as means ± SD (*n* = 6); Values with different uppercase and lowercase letters in the same column and in the same row were significantly different respectively, by Duncan’s multiple range test (*p* < 0.05); NC: normal control group; D: DOCA-salt at a dose of 20 mg/kg bw; CAP: DOCA-salt with administration of captopril at a dose of 50 mg/kg bw; DW: DOCA-salt with administration of water extract of TWK10-fermented soymilk at a dose of 2.65 g/kg bw; DE: DOCA-salt with administration of ethanol extract of TWK10-fermented soymilk at a dose of 0.09 g/kg bw.

**Table 4:** Effects of administration of the extracts from TWK10-fermented soymilk on the probe test in Morris water maze in DOCA-salt induced hypertension and its associated dementia rats.

Group	Percent Time in the Target Quadrant (s)	Target Crossing (Times)
NC	38.00 ± 10.99 ^AB^	3.50 ± 0.84 ^A^
D	27.37 ± 7.96 ^B^	1.33 ± 1.21 ^B^
CAP	35.63 ± 7.08 ^AB^	3.33 ± 1.21 ^A^
DW	36.57 ± 10.41 ^AB^	2.83 ± 1.33 ^A^
DE	42.90 ± 10.37 ^A^	3.33 ± 1.03 ^A^

Data are presented as means ± SD (*n* = 6); Values with different uppercase letters in the same column were significantly different by Duncan’s multiple range test (*p* < 0.05); NC: normal control group; D: DOCA-salt at a dose of 20 mg/kg bw; CAP: DOCA-salt with administration of captopril at a dose of 50 mg/kg bw; DW: DOCA-salt with administration of water extract of TWK10-fermented soymilk at a dose of 2.65 g/kg bw; DE: DOCA-salt with administration of ethanol extract of TWK10-fermented soymilk at a dose of 0.09 g/kg bw.

**Table 5:** Effects of administration of the extracts from TWK10-fermented soymilk on working memory task in Morris water maze in DOCA-salt induced hypertension and its associated dementia rats.

Group	Total Swimming Distance (cm)			Escapes Latency (s)		
	Day 5	Day 6	Day 7	Day 5	Day 6	Day 7
NC	273.49 ± 132.32 ^B^	193.84 ± 78.86 ^B^	210.79 ± 58.34 ^B^	9.74 ± 6.00 ^B^	6.55 ± 3.53 ^B^	6.46 ± 2.46 ^B^
D	994.17 ± 353.26 ^A^	882.38 ± 531.74 ^A^	652.35 ± 261.52 ^A^	34.40 ± 13.24 ^A^	28.14 ± 22.37 ^A^	23.86 ± 13.92 ^A^
CAP	235.19 ± 100.68 ^B^	241.74 ± 112.12 ^B^	196.03 ± 60.04 ^B^	8.22 ± 4.68 ^B^	8.86 ± 4.61 ^B^	6.86 ± 3.45 ^B^
DW	189.24 ± 81.93 ^B^	247.20 ± 136.60 ^B^	198.95 ± 91.06 ^B^	6.96 ± 4.20 ^B^	8.49 ± 4.74 ^B^	5.99 ± 3.16 ^B^
DE	181.60 ± 78.92 ^B^	217.08 ± 107.24 ^B^	185.74 ± 70.12 ^B^	6.32 ± 3.84 ^B^	7.45 ± 3.35 ^B^	5.79 ± 2.56 ^B^

Data are presented as means ± SD (*n* = 6); Values with different uppercase letters in the same column were significantly different by Duncan’s multiple range test (*p* < 0.05); NC: normal control group; D: DOCA-salt at a dose of 20 mg/kg bw; CAP: DOCA-salt with administration of captopril at a dose of 50 mg/kg bw; DW: DOCA-salt with administration of water extract of TWK10-fermented soymilk at a dose of 2.65 g/kg bw; DE: DOCA-salt with administration of ethanol extract of TWK10-fermented soymilk at a dose of 0.09 g/kg bw.

**Table 6:** Effect of administration of the extracts of TWK10-fermented soymilk on superoxide dismutase, acetylcholinesterase, catalase activities and malondialdehyde, glutathione concentration in brain of DOCA-salt hypertension induced vascular dementia rats.

Group	SOD (U/g Tissue)	MDA (nmol/g Tissue)	AChE (mU/g Tissue)	CAT (mU/g Tissue)	GSH (nmol/g Tissue)
NC	18.01 ± 3.92 ^A^	16.45 ± 4.59 ^D^	503.87 ± 44.12 ^C^	973.88 ± 35.94 ^A^	139.62 ± 27.41 ^A^
D	5.94 ± 1.53 ^C^	32.33 ± 3.88 ^A^	566.50 ± 13.88 ^A^	832.35 ± 125.74 ^B^	106.55 ± 7.75 ^B^
CAP	8.16 ± 0.84 ^BC^	28.00 ± 4.20 ^AB^	546.34 ± 13.42 ^AB^	1004.97 ± 57.53 ^A^	140.44 ± 23.08 ^A^
DW	10.83 ± 2.58 ^B^	23.67 ± 3.20 ^BC^	520.01 ± 26.81 ^BC^	958.13 ± 90.33 ^A^	114.31 ± 13.58 ^AB^
DE	18.58 ± 1.64 ^A^	20.00 ± 4.38 ^CD^	530.25 ± 22.23 ^BC^	957.17 ± 119.58 ^A^	120.79 ± 39.51 ^AB^

Data are presented as means ± SD (*n* = 6); Values with different uppercase letters in the same column were significantly different by Duncan’s multiple range test (*p* < 0.05); AChE: acetylcholinesterase; CAT: catalase; GSH: glutathione SOD: superoxide dismutase; MDA: malondialdehyde; NC: normal control group; D: DOCA-salt at a dose of 20 mg/kg bw; CAP: DOCA-salt with administration of captopril at a dose of 50 mg/kg bw; DW: DOCA-salt with administration of water extract of TWK10-fermented soymilk at a dose of 2.65 g/kg bw; DE: DOCA-salt with administration of ethanol extract of TWK10-fermented soymilk at a dose of 0.09 g/kg bw.

**Table 7:** Effects of administration of the extracts of TWK10-fermented soymilk on ACE activity in lung and kidney, angiotensin II and nitric oxide concentration in plasma of DOCA-salt induced hypertension and its associated dementia rats.

Group	ACE Activity in Lung (U/mL)	ACE Activity in Kidney (U/mL)	Angiotensin II (ng/mL)	Nitrate + Nitrite (μM)
NC	17.81 ± 4.84 ^BC^	14.22 ± 2.21 ^BC^	0.22 ± 0.04 ^B^	31.27 ± 1.46 ^B^
D	29.64 ± 3.51 ^A^	18.11 ± 3.94 ^A^	0.29 ± 0.04 ^A^	25.55 ± 1.89 ^C^
CAP	12.47 ± 8.26 ^C^	11.16 ± 2.84 ^C^	0.21 ± 0.03 ^B^	30.99 ± 1.15 ^B^
DW	19.81 ± 3.77 ^B^	15.68 ± 2.69 ^AB^	0.20 ± 0.06 ^B^	31.72 ± 1.97 ^B^
DE	22.44 ± 5.67 ^B^	16.24 ± 2.43 ^AB^	0.22 ± 0.06 ^B^	34.10 ± 0.33 ^A^

Data are presented as means ± SD (*n* = 6); Values with different uppercase letters in the same column were significantly different by Duncan’s multiple range test (*p* < 0.05); NC: normal control group; D: DOCA-salt at a dose of 20 mg/kg bw; CAP: DOCA-salt with administration of captopril at a dose of 50 mg/kg bw; DW: DOCA-salt with administration of water extract of TWK10-fermented soymilk at a dose of 2.65 g/kg bw; DE: DOCA-salt with administration of ethanol extract of TWK10-fermented soymilk at a dose of 0.09 g/kg bw.

## Abbreviations

The following abbreviations are used in this manuscript:

AD	Alzheimer’s disease
VaD	vascular dementia
DOCA	deoxycorticosterone acetate
MWM	Morris water maze
BP	blood pressure
ACE	angiotensin-converting enzyme
CAP	captopril
HHL	hippuryl-l-histidyl-l-leucine
FAPGG	*N*-[3-(2-furyl) acryloyl]-l-phenylalanylglycyl-glycine
AsA	l-ascorbic acid
MTT	3-[4,5-dimethylthiazol-2-yl]-2,5-diphenyltetrazolium bromide
DMSO	dimethyl sulfoxide
DPPH	α,α-diphenyl-β-picrylhydrazyl
NGF	nerve growth factor
SBP	systolic blood pressure
DBP	diastolic blood pressure
TBARS	thiobarbituric acid reactive substance
NO	nitrous oxide
